# The effects of a dopamine agonist (apomorphine) on experimental and spontaneous pain in patients with chronic radicular pain: A randomized, double-blind, placebo-controlled, cross-over study

**DOI:** 10.1371/journal.pone.0195287

**Published:** 2018-04-05

**Authors:** May Haddad, Dorit Pud, Roi Treister, Erica Suzan, Elon Eisenberg

**Affiliations:** 1 Faculty of Social Welfare and Health Sciences, University of Haifa, Haifa, Israel; 2 Institute of Pain Medicine, Rambam Health Care Campus, Haifa, Israel; 3 The Rappaport Faculty of Medicine, Technion–Israel Institute of Technology, Haifa, Israel; University of Würzburg, GERMANY

## Abstract

**Background:**

Although evidence suggests that dopaminergic systems are involved in pain processing, the effects of dopaminergic interventions on pain remains questionable. This randomized, double blinded, placebo-controlled, cross-over study was aimed at exploring the effect of the dopamine agonist apomorphine on experimental pain evoked by cold stimulation and on spontaneous pain in patients with lumbar radicular (neuropathic) pain.

**Methods:**

Data was collected from 35 patients with chronic lumbar radiculopathy (18 men, mean age 56.2±13 years). The following parameters were evaluated before (baseline) and 30, 75 and 120 minutes subsequent to a subcutaneous injection of 1.5 mg apomorphine or placebo: cold pain threshold and tolerance in the painful site (ice pack, affected leg) and in a remote non-painful site (12°C water bath, hand), and spontaneous (affected leg) pain intensity (NPS, 0–100).

**Results:**

One-hundred and twenty minutes following apomorphine (but not placebo) injection, cold pain threshold and tolerance in the hand increased significantly compared to baseline (from a median of 8.0 seconds (IQR = 5.0) to 10 seconds (IQR = 9.0), p = 0.001 and from a median of 19.5 seconds (IQR = 30.2) to 27.0 seconds (IQR = 37.5), p<0.001, respectively). In addition, apomorphine prolonged cold pain tolerance but not threshold in the painful site (from a median of 43.0 seconds (IQR = 63.0) at baseline to 51.0 seconds (IQR = 78.0) at 120 min, p = 0.02). Apomorphine demonstrated no superiority over placebo in reducing spontaneous pain intensity.

**Conclusion:**

These findings are in line with previous results in healthy subjects, showing that apomorphine increases the ability to tolerate cold pain and therefore suggesting that dopaminergic interventions can have potential clinical relevance.

## Introduction

Evidence shows that dopaminergic systems are involved in central pain processing. Dopaminergic neurotransmission can modulate pain perception by acting at supraspinal regions including the basal ganglia [1], insula [2], anterior cingulated cortex [3], and periaquaductal gray [4], as well as within the spinal cord [5]. Furthermore, in addition to opioids and other cathecolamines, dopamine seems to play a role in descending pain inhibition [5–7].

In a study conducted in our laboratory, the dopamine agonist apomorphine significantly prolonged tolerance to experimental cold pain in healthy volunteers [8]. Yet, other studies failed to show similar effects on experimental pain: In one study, dopamine precursor depletion failed to attenuate brief thermal pain induced by laser stimulation [9]. In another study, a transient decrease of dopamine activity by either dopamine precursor depletion or by administrating the D2-receptor antagonist sulpiride, had no effects on thermal pain thresholds, tolerance, or temporal summation [10].

From the clinical standpoint, a limited number of trials support the idea that dopaminergic interventions can change various aspects of pain perception. Such changes have been reported in clinical conditions associated with abnormalities in dopaminergic neurotransmission such as Parkinson's disease, fibromyalgia, restless leg syndrome and burning mouth syndrome. In patients with Parkinson’s disease, L-DOPA increased pain thresholds [11–13] and the dopamine agonist rotigotine attenuated spontaneous pain [14]. In fibromyalgia patients, fatigue and function improved in response to the administration of the dopamine agonist Pramipexole [15]. Pramipexole also decreased pain intensity in patients with burning mouth syndrome [16]. Improvement in clinical pain in response to several dopamine agonists has also been reported in patients with restless legs [17–19].

Importantly, a limited number of small clinical trials showed evidence for the efficacy of dopaminergic manipulations in patients with various forms of neuropathic pain (NP). A significant decrease in pain intensity was demonstrated in patients with herpes zoster following levodopa administration but not placebo [20]. In another study, the administration of levodopa significantly reduced NP intensity in comparison to placebo in patients with painful diabetic neuropathy [21]. To date, evidence is inconsistent, and no firm conclusions regarding the efficacy of dopaminergic agents for the treatment of NP can be drawn. At the same time, for the majority of patients with NP analgesic treatments provide only partial pain relief, thus creating a crucial need to identify new effective pharmacological interventions for treating NP [22–24].

The possible effect of dopaminergic manipulations on NP, the presence of cold hyperalgesia/allodynia in many patients with NP [25,26] and also the effect of dopaminergic manipulations on experimental cold pain, laid the ground for the present translational study. In the present study, we aimed to investigate the effect of apomorphine on experimental cold pain and on spontaneous pain intensity in patients with NP. We hypothesized that apomorphine will prolong cold pain tolerance at the non-painful site (i.e., *experimentally* evoked cold pain) as well as at the painful site (i.e., *clinically* evoked cold pain) and possibly affect spontaneous pain intensity as well.

## Methods

### Ethical aspects

The study was conducted in accordance with Good Clinical Practice (GCP) guidelines and was approved on July 19, 2011 by the Rambam Health Care Campus Ethics Committee (RMB-234-11). Signed written informed consent was obtained from all patients following a detailed explanation regarding the study's purpose and procedures. The study was registered in the ClinicalTrials.gov Protocol Registration System, registration number NCT02969629. Notably, the study was registered only after patient recruitment began since the local Helsinki committee did not require registration at that time. The authors confirm that all ongoing and related trials for this drug/intervention are registered.

### Patients

Patients with chronic lumbar radiculopathy were recruited from the Pain Relief Institute in Rambam Health Care Campus and by advertising in local newspapers. Patients were recruited between December 7, 2011 and November 27, 2013 and the study was completed on July 19, 2015.

Potential patients were invited to a screening visit at the Pain Relief Institute, for the diagnosis of lumbar radicular NP according to the IASP recommended criteria. Accordingly, diagnosis was based on patients' report of pain, physical and neurological examinations and review of imaging tests [27]. Patients were eligible for enrollment in the study if the following inclusion criteria were met: 1) Presence of lumbar radicular pain for at least 3 months; 2) Average pain level during the last week prior to enrollment >40 (Numerical Pain Scale ranging from 0 to 100); 3) No use of a new analgesic drug within 30 days prior to entry to the study; and 4) Adults who were capable of understanding the purpose and instructions of the study and signing an informed consent. Exclusion criteria were: 1) Pregnancy or breastfeeding; 2) Presence of Parkinson's disease or any other extra-pyramidal diseases; 3) History of allergy to the investigational drugs: Apomorphine or Domperidone; 4) History of polyneuropathy; and 5) Respiratory depression, dementia, psychiatric diseases or hepatic insufficiency.

**Fig 1:**
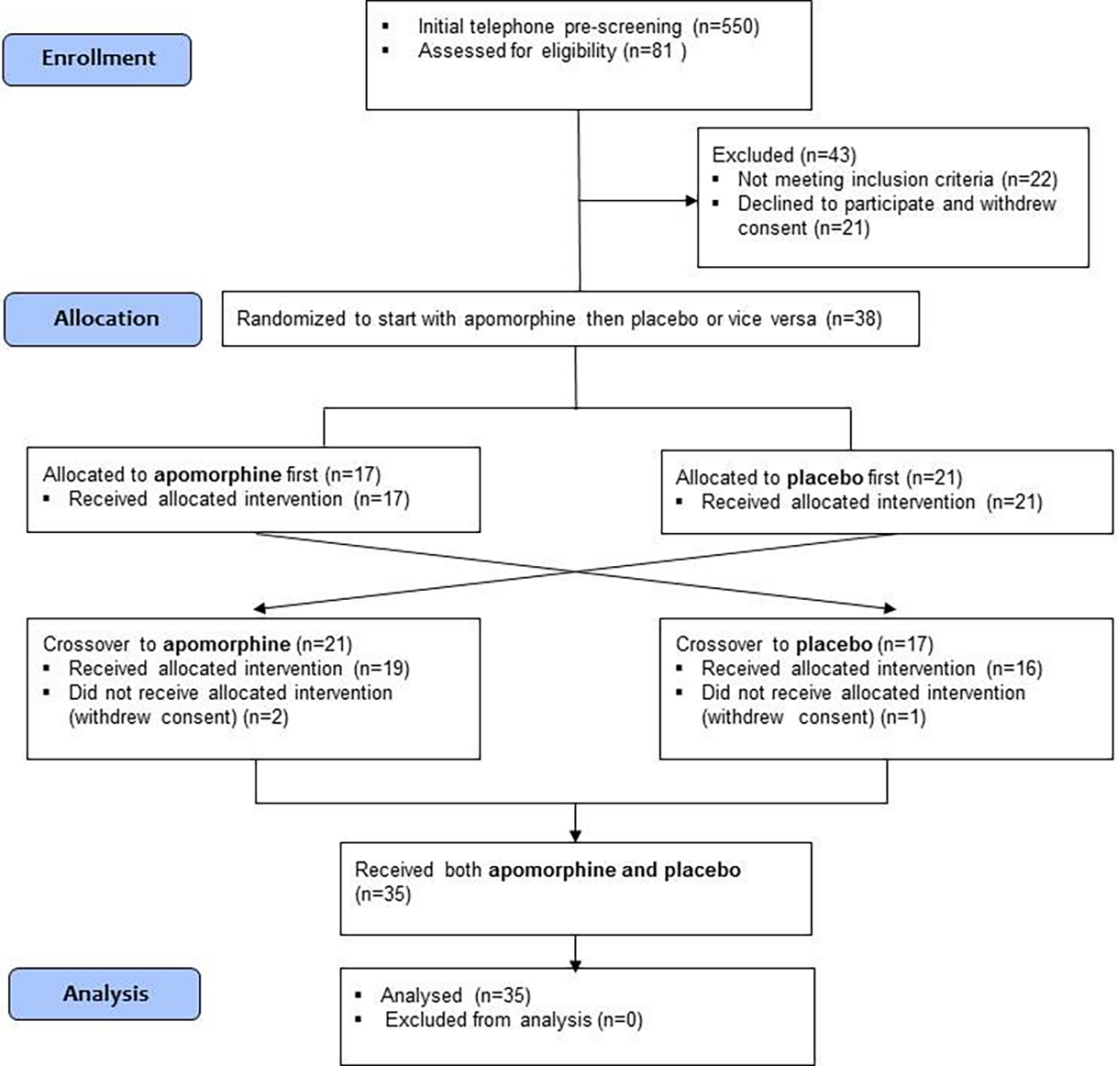
Patients’ recruitment flowchart.

The patients' recruitment process is depicted in Fig 1. As can be seen, 550 potential patients were contacted. Eighty one patients were invited to a screening visit in the Pain Relief Institute and fifty-nine patients were found eligible and signed an informed consent form. Thirty-eight patients entered the study. Three patients participated in one session only. Hence, the per-protocol population comprised of thirty-five patients.

### Instruments and pain measures

#### Experimental cold pain evoked by noxious cold stimulation (non-painful site)

The Cold Pressor Test (CPT) apparatus (Heto CBN 8–30 Lab equipment, Allerod, Denmark) was used for assessment of cold pain perception in the hand. This apparatus is a temperature-controlled water bath with a maximum temperature variance of ± 0.5°C, which is continuously stirred by a pump. Patients were asked to place their dominant hand in the CPT apparatus (12°C) in a still position while their fingers spread wide apart. A stopwatch was simultaneously activated, and patients were requested to maintain their hand in the cold water for as long as they could. They were instructed to indicate the exact point in time when the cold sensation began to elicit pain and this time was recorded as the threshold of cold pain (seconds) and was considered as the secondary outcome measure. Time until spontaneous hand withdrawal was recorded as cold pain tolerance (seconds). A cut-off time of 180 seconds was set for safety reasons. The cold pain tolerance was regarded as the primary outcome of the study.

#### Cold pain evoked by noxious cold stimulation (painful site)

A flexile ice pack (25 cm X 17 cm) was placed on the area of maximal pain intensity in the affected leg. This maneuver was aimed at evoking the subjects’ clinical pain. A stopwatch was simultaneously activated, and patients were requested to keep the ice pack on the leg for as long as they could. The patients were instructed to indicate the exact time point when the cold sensation began to elicit pain (cold pain threshold; seconds). The cold pain threshold was considered as the secondary outcome measure. Time until the ice pack was not tolerated anymore was also recorded and defined as clinical evoked cold pain tolerance (seconds). A cut-off time of 180 seconds was set for safety reasons. The clinical evoked cold pain tolerance was regarded as an additional primary outcome of the study.

#### Numerical pain scale (NPS)

The numerical pain scale (NPS) ranging from 0 = ‘‘no pain” to 100 = ‘‘the worst pain one can imagine”, was verbally used by patients to rate the magnitude of their current spontaneous pain intensity in the area of maximal pain intensity in the affected leg. NPS was considered as the secondary outcome measure.

### Study medications

The study medications included: 1) a single dose of apomorphine, an injectable, potent, short-acting dopamine agonist. It is administered subcutaneously and has a bio-availability of 100% which assures considerable short-lived dopamine excitation [28]. Based on a previous study conducted in our laboratory, 1.5 mg apomorphine was determined to be the appropriate dose for this study [8]; 2) An identical looking placebo (saline); 3) Domperidone, a peripheral dopamine antagonist aimed to reduce apomorphine side effects.

Apomorphine and the identical looking placebo (saline) syringes were prepared and injected by a nurse, who was not a part of the research team, according to a pre-determined randomization. Randomization of the order of apomorphine and placebo administration was done according to a computer-generated random code.

### Study design

Patients diagnosed with chronic lumbar radiculopathy who met the inclusion criteria were enrolled in this randomized, double-blind, placebo-controlled, cross-over study. Each eligible subject received detailed information about the study and its procedures and an informed consent was obtained. Following enrollment, each subject participated in the two study sessions that took place about one week apart. Each in-clinic session lasted three hours. Patients were instructed to take domperidone tablets, 3 times a day, for 3 days before each session.

At each session, patients were instructed to stay in supine position throughout the entire session. Pain tests were performed in the following order: current spontaneous neuropathic pain in the area of maximal pain intensity in the affected leg; cold pain threshold and tolerance in a non-painful site (hand); cold pain threshold and tolerance in the most painful site in the affected leg. A first battery of tests was performed and considered as training. Fifteen minutes later a second battery was conducted and the results were recorded and regarded as baseline measurements.

**Fig 2:**
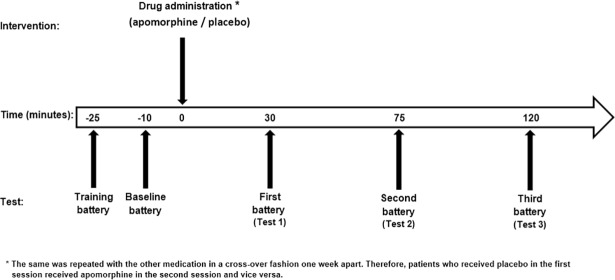
Study design.

Ten minutes following baseline measures, patients received either 1.5 mg subcutaneous apomorphine or an identical looking placebo (saline) in a double blind fashion. The experimenter was blinded to both the injected drug (apomorphine/placebo) and to the subjects’ adverse effect reports. Three additional test batteries were conducted 30, 75, and 120 minutes after drug administration (test 1, test 2 and test 3, respectively; Fig 2). These time points were chosen based on results from a previous study conducted in our laboratory [8]. One week later, a second session was conducted in the same manner with the administration of the other medication (apomorphine or placebo).

During each session, patients were given a form that included most common drug side effects and were asked to self-report side effects experienced 10, 55, 100 and 150 minutes following drug administration. These were reported on a 0–3 scale, where 0 = none, 1 = mild, 2 = moderate, and 3 = severe. The following side effects were monitored: sweating, breathing difficulties, dry mouth, drowsiness, headache, dizziness, nausea/vomiting, confusion, itch, blushing and diarrhea. Subjects were instructed not to discuss their adverse effects with the experimenter unless perceived as severe.

### Statistical analyses

The sample size for this between and within analysis, with two treatment groups and four repeated measurements design was calculated by G* Power 3.1 [29]. The sample size was calculated according to a medium effect size (f = 0.30) to achieve appropriate statistical power (β = 0.80), with the acceptable two-sided type 1 error (α = 0.05). The required sample with the aforementioned assumptions was found to be 35 subjects.

All analyses were conducted using the SPSS for Windows Version 21 statistical package (SPSS, Inc., Chicago, IL). Pain measures and demographics at screening are presented as mean±standard deviation (SD) as well as medians. Descriptive statistics were generated for all pain measures and all results are presented as medians and interquartile range (IQR) in tables. In addition, the 25th and 75th percentiles were calculated and presented as boxplots. As pain measures were not normally distributed, non-parametric tests for dependent samples (Friedman Chi-square test) were conducted to evaluate differences in pain measures among the different time points following drug administration (apomorphine or placebo). Results were considered significant only if a significant change from baseline in any given parameter was found following administration of apomorphine but not placebo. Follow-up pairwise comparisons were conducted using Wilcoxon signed–rank test to identify significant differences between two specific time points for each pain measure. The possibility that the findings are the result of a type I statistical error cannot be discounted. Thus, Bonferroni correction was applied, and results were considered significant at the p = 0.01 level (0.05/5). Effect sizes were calculated as the absolute value of the Z score divided by the square root of n.

## Results

### Patients

As shown in Fig 1, data was collected from thirty-five patients (17 women and 18 men). Their ages ranged from 23 to 78 (mean ± SD: 56.2±13.1). Pain duration ranged from 4 to 240 months (mean ± SD = 54.1±61.2 months; median = 36.0). Pain intensity (NPS 0–100) at the screening visit ranged from 40 to 90 (mean ± SD = 68.8±13.0; median = 70.0).

### Baseline measurements

Comparisons of baseline measurements between placebo and apomorphine sessions were conducted (2-tailed T-test) and revealed no significant differences between the two conditions in any of the tested measures.

### Apomorphine effect on experimentally evoked cold in a remote non-painful site (hand)

**Fig 3:**
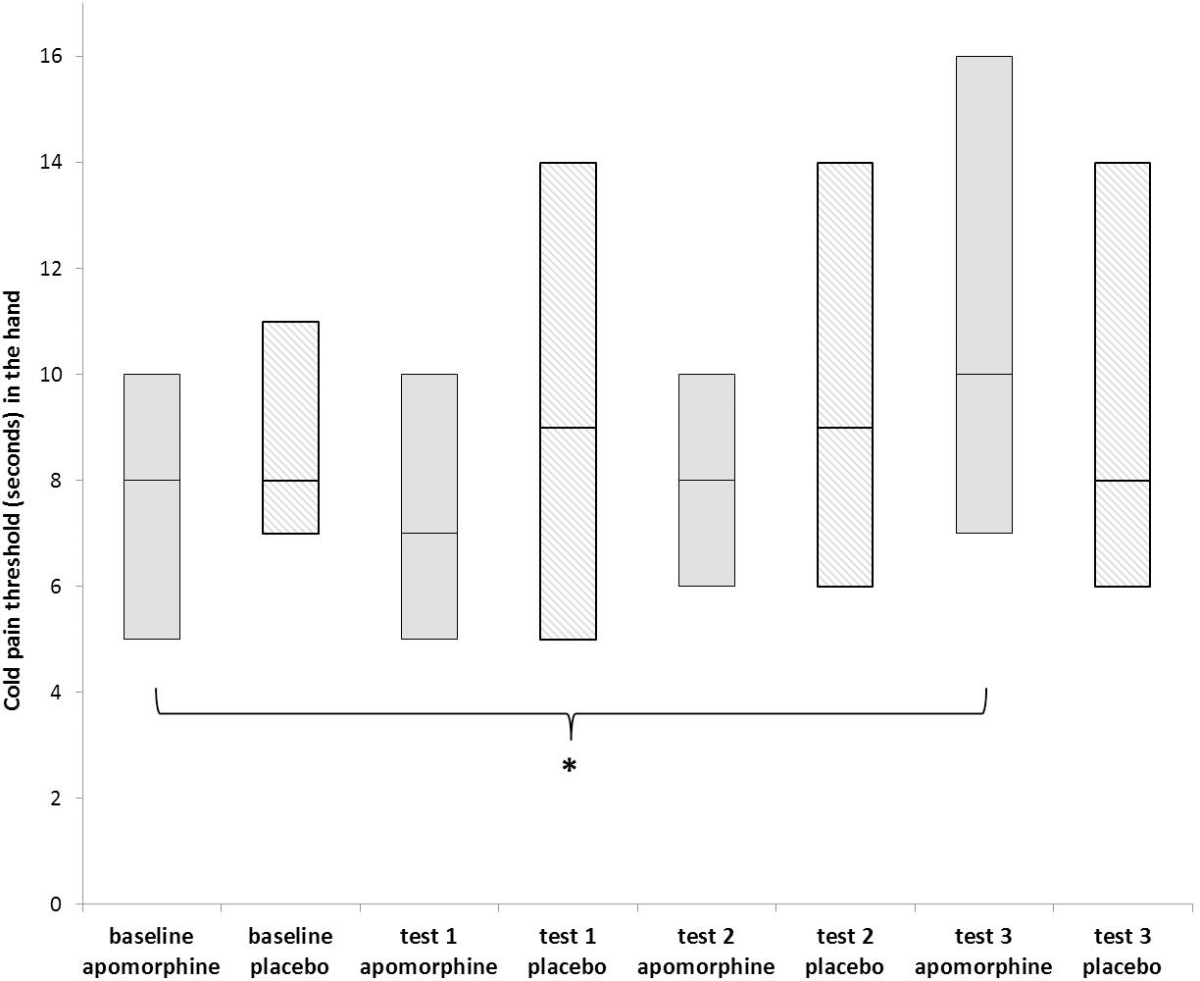
Apomorphine/placebo effect on cold pain threshold (seconds) in a non-painful site (hand) (n = 31). Boxplot representing median, 25^th^ and 75^th^ percentiles for cold pain threshold (sec) in the hand for the two study conditions, measured before (baseline) and after drug administration. The asterisk represents a significant difference only between baseline and test 3 measurements following apomorphine administration. * <0.01.

As demonstrated in Table 1 and Fig 3, cold pain threshold increased significantly following the administration of apomorphine (Friedman test, Chi-Square = 16.70; p = 0.001) and Wilcoxon analyses showed a significant increase in threshold from a median of 8.0 sec (IQR = 5.0) at baseline to 10 sec (IQR = 9.0) 120 minutes after drug administration (test 3) (Wilcoxon test, p = 0.001; effect size = 0.42). As expected, no significant changes were found in cold pain threshold following placebo administration (Friedman test, Chi-Square = 2.03; p = 0.56).

**Table 1:** Apomorphine/placebo effects on experimental cold pain in a non-painful site (hand).

Pain measure	Medication	Baseline	Test 1	Test 2	Test 3	Friedman test	Wilcoxon test
**Cold pressor test (CPT) Threshold (sec)**	apomorphine	8.0, 5.0	7.0, 5.0	8.0, 4.0	10.0, 9.0	χ^2^_(3, 31)_ = 16.70 p = 0.001	Test 3>baseline p = 0.001
	placebo	8.0, 4.0	9.0, 9.0	9.0, 8.0	8.0, 8.0	χ^2^_(3, 35)_ = 2.03 p = 0.56	
**Cold pressor test (CPT) Tolerance (sec)**	apomorphine	19.5, 30.2	20.0, 35.0	21.5, 27.5	27.0, 37.5	χ^2^_(3, 28)_ = 23.92 p<0.001	Test 3>baseline p<0.001
	placebo	22.0, 21.0	22.0, 20.0	22.0, 20.0	21.0, 23.0	χ^2^_(3, 31)_ = 1.42 p = 0.70	

Pain measures are shown as median, IQR

**Fig 4:**
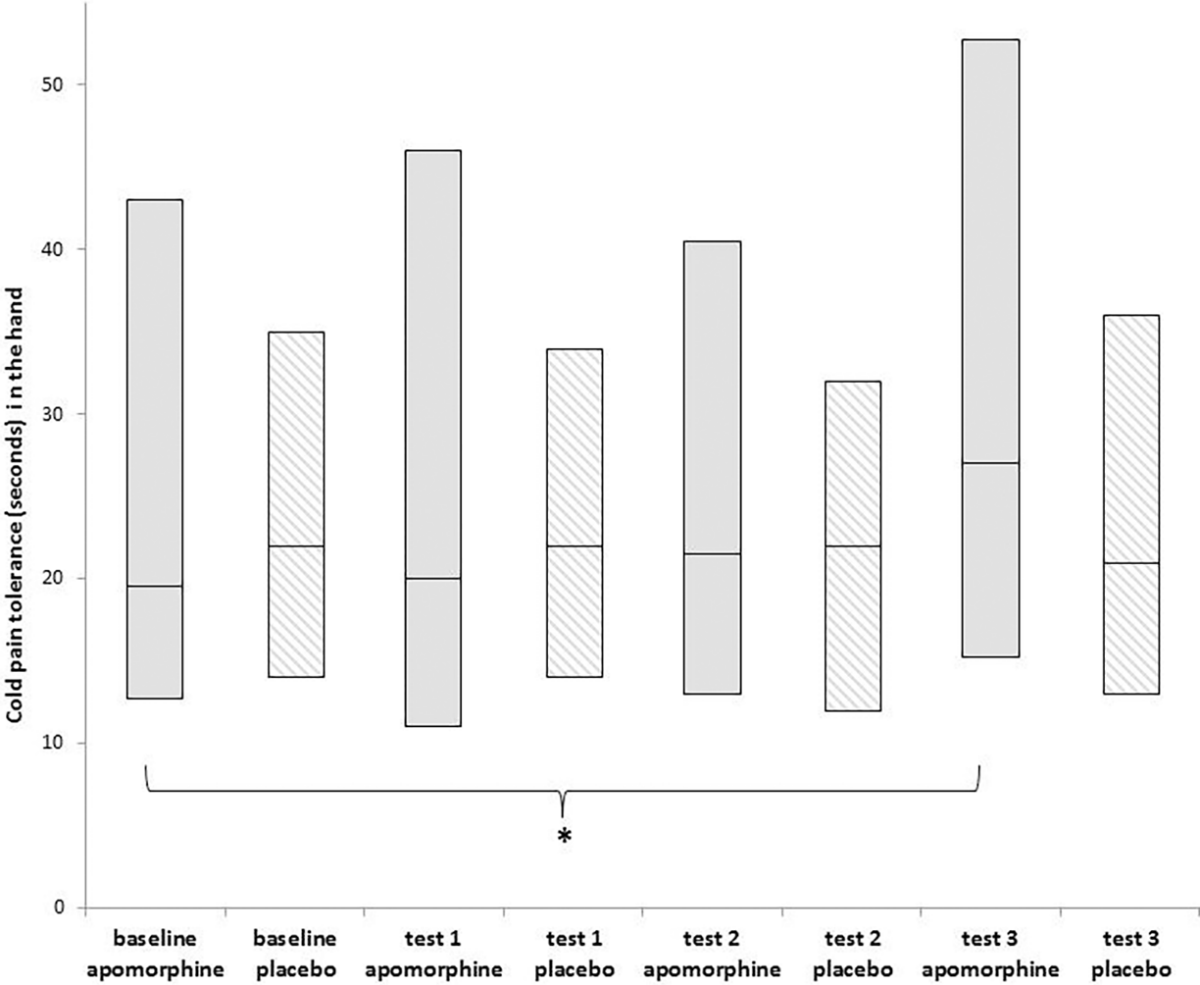
Apomorphine/placebo effect on cold pain tolerance (seconds) in a non-painful site (hand) (n = 28). Boxplot representing median, 25th and 75th percentiles for cold pain tolerance (sec) in the hand for the two study conditions, measured before (baseline) and after drug administration. The asterisk represents a significant difference only between baseline and test 3 measurements following apomorphine administration. * <0.01.

In addition, cold pain tolerance increased significantly following the administration of apomorphine (Friedman test, Chi-Square = 23.92; p<0.001) and the Wilcoxon analyses showed a significant increase in tolerance from a median of 19.5 sec (IQR = 30.2) at baseline to 27.0 sec (IQR = 37.5) 120 minutes after drug administration (test 3) (Wilcoxon test, p<0.001; effect size = 0.53). No significant changes were found in cold pain tolerance following placebo administration (Friedman test, Chi-Square = 1.42; p = 0.70) (Table 1; Fig 4). Notably, four patients exceeded the cut-off point of 180 seconds at baseline and therefore were excluded from the CPT tolerance analyses.

### Apomorphine effect on cold pain (affected leg) evoked by noxious cold stimulation

**Fig 5:**
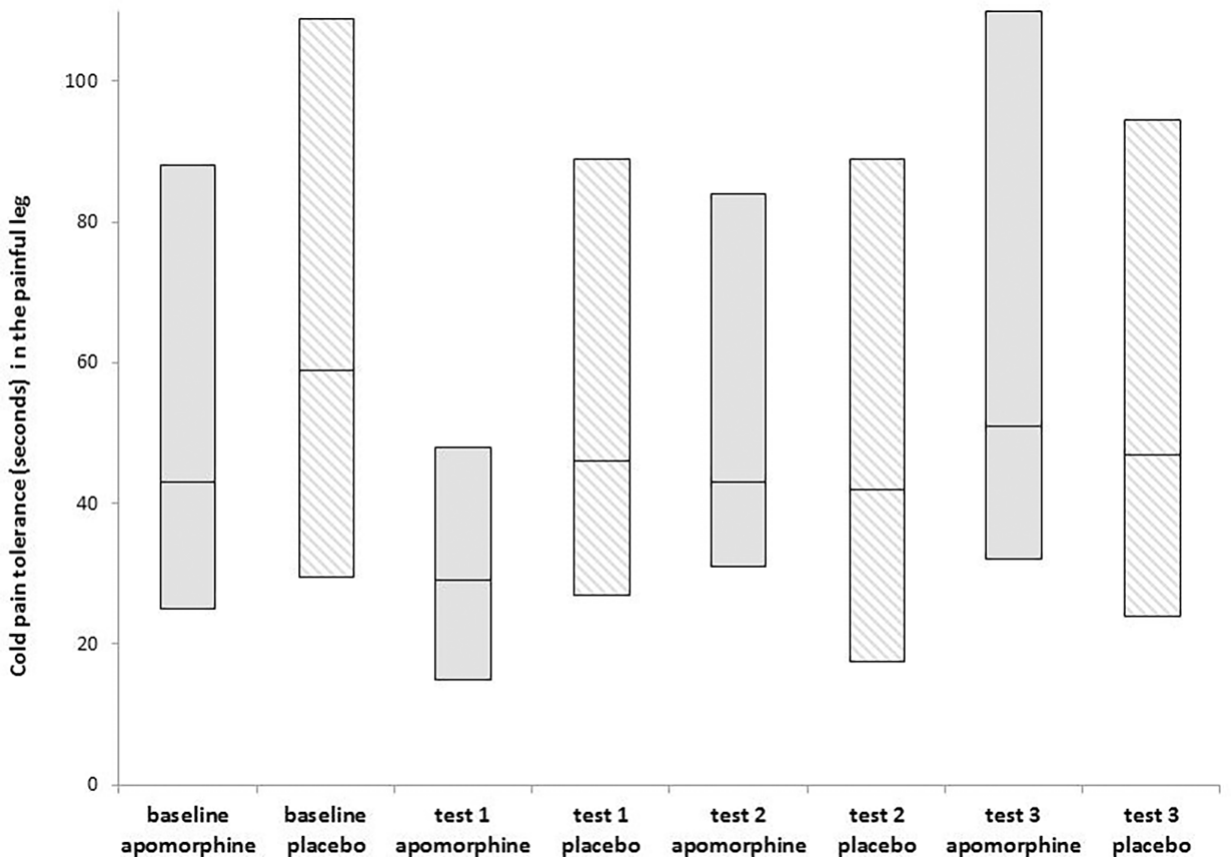
Apomorphine/placebo effect on cold pain tolerance in the painful leg (n = 19). Boxplot representing median, 25th and 75th percentiles for cold pain tolerance (sec) in the affected leg for the two study conditions, measured before (baseline) and after drug administration. No significant differences between baseline and test 1–3 measurements were detected following apomorphine/placebo administration.

While exposing the patients to the ice packs, 34 out of the 35 patients reported that the application was painful. However, 14 of the patients exceeded the cut-off point of 180 seconds at baseline and were excluded from the cold pain tolerance analyses. As shown in Table 2 and Fig 5, in the remaining patients, at test 3, cold pain tolerance in the affected leg increased from a median of 43.0 sec (IQR = 63.0) at baseline to 51.0 sec (IQR = 78.0). This increase, however, had only a trend of significance (Friedman test, Chi-Square = 9.61; p = 0.02). No significant changes were found in cold pain tolerance following placebo administration (Friedman test, Chi-Square = 1.72; p = 0.63).

**Table 2:** Apomorphine/placebo effects on spontaneous pain and on pain evoked by cold stimulation in the painful leg.

Pain measure	Medication	Baseline	Test 1	Test 2	Test 3	Friedman test
**Cold (ice) pain test Threshold (sec)**	apomorphine	18.0, 23.0	17.0, 29.0	21.0, 33.0	23.0, 37.0	χ^2^_(3, 31)_ = 19.90 p<0.01
	placebo	15.0, 16.7	17.0, 21.5	19.0, 21.2	18.0, 33.0	χ^2^_(3, 34)_ = 10.25 p = 0.01
**Cold (ice) pain test Tolerance (sec)**	apomorphine	43.0, 63.0	29.0, 33.0	43.0, 53.0	51.0, 78.0)	χ^2^_(3, 19)_ = 9.61 p = 0.02
	placebo	59.0, 79.5	46.0, 62.0	42.0, 71.5	47.0, 70.5	χ ^2^_(3, 21)_ = 1.72 p = 0.63
**Spontaneous pain** **(0–100)**	apomorphine	60.0, 25.0	45.0, 50.0	30.0, 37.5	30.0, 40.0	χ^2^_(3, 33)_ = 51.82 p<0.001
	placebo	60.0, 40.0	40.0, 40.0	40.0, 40.0	30.0, 40.0	χ^2^_(3, 35)_ = 30.25 p<0.001

Pain measures are shown as median, IQR

Significant changes in threshold to cold pain in the affected leg following both apomorphine (Friedman test, Chi-Square = 19.90; p<0.01) and placebo (Friedman test, Chi-Square = 10.25; p = 0.01) administration were detected. The significant change from baseline under the two conditions (apomorphine and placebo), precludes a specific effect of apomorphine on cold pain threshold in the most painful site.

### Apomorphine effect on spontaneous pain intensity

Before apomorphine administration, the median (IQR) baseline spontaneous pain intensity was 60.0 (25.0). Thirty, 75 and 120 minutes after drug administration (test 1, 2, 3 respectively), pain decreased gradually and significantly to 45 (50), 30.0 (37.5) and 30 (40.0), respectively (Friedman test, Chi-Square = 51.82; p>0.001). However, a significant decrease in spontaneous pain was also found following placebo administration from 60 (40.0) to 40 (40), 40 (40) and 30 (40) (NPS), 30, 75 and 120 minutes after drug administration, respectively (Chi-Square = 30.25; p<0.001). Post hoc Wilcoxon analyses showed a significant decrease in spontaneous pain in all time points following both drug administrations, as compared to baseline (Wilcoxon test, p<0.001 for all measurements) (Table 2). As such, the effect of apomorphine on spontaneous pain is still questionable.

Notably, due to side effects, some tests could not be completed by all patients. Therefore the number of patients who completed each test is noted next to each tested measure in the tables and figures.

### Side effects

Ten minutes following apomorphine administration, 77% of the patients reported at least one side effect compared to 17% following placebo administration. These percentages dropped to 71% and 14%, respectively, after 55 minutes, and to 31% and 14%, after 100 minutes. The most common side effect caused by apomorphine was drowsiness whereas headache was the most frequent side effect following placebo. The side effects experienced by patients throughout all time points are summarized in Table 3. All side effects resolved spontaneously at the 150 min follow-up time point. No serious adverse effects were noted.

**Table 3:** The number of patients who experienced different side effects.

	Apomorphine				Placebo			
	none	mild	moderate	severe	none	mild	moderate	severe
**Sweating**	31	1	3	0	35	0	0	0
**Breathing difficulties**	34	1	0	0	33	1	1	0
**Dry mouth**	30	2	2	1	32	2	1	0
**Drowsiness**	7	12	11	5	32	2	1	0
**Headache**	32	2	1	0	30	3	1	1
**Dizziness**	21	10	3	1	32	2	1	0
**Nausea/ vomiting**	25	7	0	3	32	2	1	0
**Mood swings**	34	1	0	0	35	0	0	0
**Confusion**	31	3	0	1	34	1	0	0
**Itch**	35	0	0	0	35	0	0	0
**Blushing**	34	0	1	1	34	1	0	0
**Diarrhea**	35	0	0	0	35	0	0	0

## Discussion

The present study demonstrated that the dopamine agonist apomorphine, but not a placebo, had a prolonging effect on experimental evoked cold pain threshold and tolerance tested in a remote, non-painful site in subjects with lumbar radicular pain. In the most painful site, apomorphine showed a trend for prolonging the tolerance to cold induced pain, but had no effect on cold pain threshold. In addition, apomorphine had no advantage relative to placebo on spontaneous radicular pain intensity.

There are conflicting views regarding the way by which the modulating effects of dopaminergic systems on pain responses is expressed. While in some studies dopaminergic manipulations had an effect on pain intensities [14–21], in others it had an effect on mechanical or thermal pain thresholds or tolerance only [8, 11–13, 30].

When referring specifically to NP, dopamine agonists have shown to induce analgesia in two small clinical trials with NP related to herpes zoster and diabetic neuropathies [20,21]. To the best of our knowledge, over a period of nearly two decades the effects of dopaminergic manipulations on neuropathic pain have not been investigated. In the present study we have not been able to demonstrate a similar analgesic effect of apomorphine on the spontaneous radicular pain in patients with radicular neuropathic pain. This is due to the fact that significant decrease in spontaneous pain was found following the administration of both apomorphine and placebo. We thererfore believe that this reduction in spontaneous pain intensity can be attributed to the long stay in supine position throughout the sessions regardless of the injected drug. An alternative explanation is a strong placebo effect on spontaneous pain in the affected leg. Indeed, reports from recent years point to increasing placebo effects on pain intensities over the years in randomized controlled trials (RCTs) of drugs for the treatment of chronic neuropathic pain [31]. Thus, the placebo effect on the spontaneous pain intensity in our study can be a part of this universal phenomenon. Hence, firm conclusions regarding the pain reduction in response to dopaminergic interventions cannot be drawn.

Three studies from our laboratories showed that dopaminergic interventions affect mostly cold pain measures, particularly cold pain tolerance and possibly cold pain threshold, but not pain intensity. First, an increase in cold pain tolerance in healthy subjects was demonstrated subsequent to apomorphine administration but not following a placebo [8]. Second, prolongation of cold pain threshold and tolerance was found in a sample of adults with attention deficit hyperactivity disorder (ADHD) following the administration of methylphenidate (a central nervous system stimulant which primarily increases extracellular dopamine levels) in an open labeled trial [30]. Third, methylphenidate but not a placebo led to similar results in healthy subjects exposed to an experimental cold pain test [submitted]. Congruent with these findings, the present study demonstrates for the first time, a similar effect of apomorphine on experimental cold pain threshold and tolerance in patients with NP.

Moreover, since pain exacerbation in response to cold stimuli is commonly reported by patients with NP [25,26], testing the effect of a cold stimulus on the radicular pain in our patients was interesting. Due to difficulties in applying the cold pressor test strictly to the area of maximal pain intensity in the affected leg, an ice pack was put onto that area instead, and was used to test threshold and tolerance to cold induced pain. Although 34 patients reported that this maneuver induced local pain, we have not been able to demonstrate a specific extending effect of apomorphine on cold pain threshold (since both apomorphine and placebo produced similar extending effects). The lack of difference in the effect of the two interventions might be related to a relatively low intensity of the noxious cold stimulation produced by applying the ice pack compared to immersing the hand in ice cold water, where a selective effect was found. At the same time, apomorphine showed a trend for prolonging the cold pain tolerance. Since 14 patients reached the 180 second cut-off point of the cold pain tolerance test, only a small number of patients could be analyzed. A larger number could have resulted in a more significant tolerance prolongation.

The effect of apomorphine on cold pain raises a question regarding the mechanism by which dopamine can affect this type of pain. Dopaminergic manipulations can have two possible sites of action: peripheral and central. Peripherally, apomorphine might have altered the response to the cold pain stimuli due to its potential effect on skin blood flow. There is also some evidence pointing towards the involvement of peripheral dopamine receptors in nociception [32,33]. Hence, apomorphine might have affected the cold pain findings by acting peripherally. However, we assume that all such peripheral dopaminergic effects have been blocked by pre-administrating the peripheral dopamine antagonist dopmeridon prior to placebo/apomorphine injections. Regarding central dopaminergic mechanisms, cold hyperalgesia is considered as an indicator of CNS involvement in the pathogenesis of any given pain syndrome (i.e. complex regional pain syndrome or CRPS [34]. This fact, plus the well-documented central effects of dopamine agonists, suggest that the effects of apomorphine on the cold pain parameters found in our study are centrally mediated.

The prolonging effect of dopamine agonists on (cold) pain tolerance deserves further consideration. Evidence shows that tolerance represents the degree to which one is willing to continue enduring aversive stimuli. It is largely influenced by motivation which, by itself, is closely related with dopamine activity [35–37]. Neuroanatomically, the dopaminergic pathway known to be related to motivation and effort-related functions is the ventral tegmental area [38]. Therefore the changes in cold pain tolerance found in the current study, likely involve this pathway.

Noteworthy in this context is the fact that converging evidence indicates that expectations and reward also play a major role in the placebo effect [39–41]. According to the placebo-reward hypothesis, any placebo response is associated with the activation of the reward circuitry and the release of endogenous dopamine in the ventral striatum [42–44]. Thus, the involvement of dopamine in both expectations and reward (as a part of the placebo response) and in motivation (which might be the underlying cause of cold pain tolerance prolongation) can at least partially explain some of the difficulties in conducting placebo controlled trials on dopamine induced analgesia, as demonstrated in the present study. Further to that, dopamine has been known to be associated with mood alterations [45], for example depression is commonly present among patients with Parkinson's disease [46]. Depression, in term, is closely related to chronic pain [47]. Thus, dopaminergic agents can have an additional indirect effect on pain through their effect on depression.

As shown in our results, the apomorphine effect on cold pain parameters was detected 120 minutes following its administration in contrast to the motor effects of apomorphine, which appear about 10 minutes following apomorphine administration [28]. A possible explanation for this discrepancy emerges from the ‘‘tonic-phasic dopaminergic activity theory”. Tonic dopamine activity refers to levels of extra synaptic dopamine whereas phasic dopamine activity relays to synaptic response to brief bursts of neuronal firing [48]. According to this theory, tonic dopamine activity influences phasic activity so that high tonic dopamine decreases phasic dopamine release and visa-versa [49]. It has been suggested that analgesic effects of dopamine rely on the phasic dopamine system [50]. Apomorphine causes a temporal increase in tonic dopamine activity, which in turn inhibits phasic dopamine release [51]. Apomorphine peaks shortly after its administration but has a rather short half-life and therefore is present at a much lesser extent two hours after its administration. Hence, at the two-hour time point its tonic inhibition is already reduced thus allowing an increase in the phasic dopaminergic analgesic firing [52]. Notably, a similar timeframe of analgesic effect has also been reported in an earlier study with healthy volunteers exposed to experimental cold pain [8].

Some limitations should be noted: 1) since the placebo was based on saline with no active component, there were differences in the prevalence of some side effects between sessions. This may have eliminated the blinding aspect of the study by revealing the true nature of each group. The use of an active placebo that mimics the adverse effect profile of the tested drug could resolve this limitation in future studies. 2) Domperidone was administered to all patients prior to both placebo and apomorphine administration. Since evidence for involvement of peripheral dopamine receptors in nociception have been documented [32,33], we can’t rule out completely the theoretical possibility that domperidone might have had at least some blocking effect on potential peripheral analgesic effects of apomorphine. However, the evidence for peripheral involvement of dopaminergic receptors in analgesia is preclinical only [32,33] and its relevance to clinical or to experimental pain models in humans has not been tested thus far.

Lastly, a brief clinical perspective: due to the limited effectiveness of currently available treatments in reducing the intensity of many chronic painful conditions, developing other strategies for pain management is clearly required [53]. The fact that dopaminergic interventions prolonged cold pain tolerance can potentially have some clinical relevance. We cautiously suggest that interventions aimed at enhancing pain tolerance and facilitating functioning, such as apomorphine, could possibly be useful, perhaps as an adjuvant therapy, for patients with chronic pain.

## Supporting information

S1 Table CONSORT 2010 checklist. (PDF)

S1 Text Clinical trial protocol. (PDF)

S1 File Dataset. (XLS)

## References

[1] Alteir N, Stewart J. The role of dopamine in the nucleus accumbens in analgesia. Life Sci. 1999; 65(22):2269–87. 10597883 10.1016/s0024-3205(99)00298-2

[2] Burkey AR, Carstens E, Jasmin L. Dopamine reuptake inhibition in the rostral agranular insular cortex produces antinociception.J Neurosci. 1999; 19(10):4169–79. 10234044 10.1523/JNEUROSCI.19-10-04169.1999 PMC6782709

[3] Lopez-Avila A, Coffeen U, Ortega-Legaspi JM, del Angel R, Pellicer F. Dopamine and NMDA systems modulate long-term nociception in the rat anterior cingulated cortex. Pain. 2004; 111 (1–2):136–43. doi: 10.1016/j.pain.2004.06.010 15327817 10.1016/j.pain.2004.06.010

[4] Flores JA, El Banoua F, Galan-Rodriguez B, Fernandez-Espejo E. Opiate antinociception is attenuated following lesion of large dopamine neurons of the periaqueductal grey: critical role for D1 (not D2) dopamine receptors. Pain. 2004; 110(1–2):205–14. doi: 10.1016/j.pain.2004.03.036 15275769 10.1016/j.pain.2004.03.036

[5] Lindvall O, Bjorklund A, Skagerberg G. Dopamine-containing neurons in the spinal cord: anatomy and some functional aspects. Ann Neurol. 14(3):255–60, 1983 doi: 10.1002/ana.410140302 6314870 10.1002/ana.410140302

[6] Wood PB. Role of central dopamine in pain and analgesia. Expert Rev Neurother 2008; 8:781–97. doi: 10.1586/14737175.8.5.781 18457535 10.1586/14737175.8.5.781

[7] Wei H, Viisanen H, Pertovaara A. Descending modulation of neuropathic hypersensitivity by dopamine D2 receptors in or adjacent to the hypothalamic A11 cell group. Pharmacol Res. 2009; 59(5):355–63. doi: 10.1016/j.phrs.2009.01.001 19416636 10.1016/j.phrs.2009.01.001

[8] Treister R, Pud D, Ebstein RP, Eisenberg E. Dopamine transporter genotype dependent effects of apomorphine on cold pain tolerance in healthy volunteers. PLoS One. 2013; 8: e63808 doi: 10.1371/journal.pone.0063808 23704939 10.1371/journal.pone.0063808 PMC3660379

[9] Tiemann L, Heitmann H, Schulz E, Baumkötter J, Ploner M. Dopamine precursor depletion influences pain affect rather than pain sensation. PLoS One. 2014 4 23; 9(4):e96167 doi: 10.1371/journal.pone.0096167 24760082 10.1371/journal.pone.0096167 PMC3997524

[10] Becker S, Ceko M, Louis-Foster M, Elfassy NM, Leyton M, Shir Y, et al Dopamine and pain sensitivity: neither sulpiride nor acute phenylalanine and tyrosine depletion have effects on thermal pain sensations in healthy volunteers. PLoS One. 2013 11 13; 8(11):e80766 doi: 10.1371/journal.pone.0080766 24236199 10.1371/journal.pone.0080766 PMC3827462

[11] Brefel-Courbon C, Payoux P, Thalamas C, Ory F, Quelven I, Chollet F, et al Effect of levodopa on pain threshold in Parkinson’s disease: a clinical and positron emission tomography study. Mov Disord 2005; 20:1557–63. doi: 10.1002/mds.20629 16078219 10.1002/mds.20629

[12] Gerdelat-Mas A, Simonetta-Moreau M, Thalamas C, Ory-Magne F, Slaoui T, Rascol O, et al Levodopa raises objective pain threshold in Parkinson’s disease: a RIII reflex study. J Neurol Neurosurg Psychiatry 2007; 78(10):1140–2. doi: 10.1136/jnnp.2007.120212 17504881 10.1136/jnnp.2007.120212 PMC2117570

[13] Slaoui T, Mas-Gerdelat A, Ory-Magne F, Rascol O, Brefel-Courbon C. Levodopa modifies pain thresholds in Parkinson’s disease patients. Rev Neurol (Paris) 2007; 163:66–71. 17304174 10.1016/s0035-3787(07)90356-2

[14] Kassubek J, Chaudhuri KR, Zesiewicz T, Surmann E, Boroojerdi B, Moran K, et al Rotigotine transdermal system and evaluation of pain in patients with Parkinson's disease: a post hoc analysis of the RECOVER study. BMC Neurol. 2014 3 6; 14:42 doi: 10.1186/1471-2377-14-42 24602411 10.1186/1471-2377-14-42 PMC4016269

[15] Holman AJ, Myers RR. A randomized, double-blind, placebo-controlled trial of pramipexole, a dopamine agonist, in patients with fibromyalgia receiving concomitant medications. Arthritis Rheum 2005; 52:2495–505. doi: 10.1002/art.21191 16052595 10.1002/art.21191

[16] Stuginski-Barbosa J, Rodrigues GG, Bigal ME, Speciali JG: Burning mouth syndrome responsive to pramipexol. J Headache Pain 2008; 9(1):43–5. doi: 10.1007/s10194-008-0003-4 18219443 10.1007/s10194-008-0003-4 PMC3476177

[17] Conti CF, de Oliveira MM, Andriolo RB, Saconato H, Atallah AN, Valbuza JS, et al Levodopa for idiopathic restless legs syndrome: evidence-based review. Mov Disord. 2007 10 15; 22(13):1943–51. Review. doi: 10.1002/mds.21662 17659645 10.1002/mds.21662

[18] Inoue Y1, Shimizu T, Hirata K, Uchimura N, Ishigooka J, Oka Y, et al Rotigotine Trial Group. Efficacy and safety of rotigotine in Japanese patients with restless legs syndrome: a phase 3, multicenter, randomized, placebo-controlled, double-blind, parallel-group study. Sleep Med. 2013 11; 14(11):1085–91. doi: 10.1016/j.sleep.2013.07.007 24055212 10.1016/j.sleep.2013.07.007

[19] Hornyak M, Trenkwalder C, Kohnen R, Scholz H. Efficacy and safety of dopamine agonists in restless legs syndrome. Sleep Med 2012; 13(3):228–36. doi: 10.1016/j.sleep.2011.09.013 22281001 10.1016/j.sleep.2011.09.013

[20] Kernbaum S, Hauchecorne J. Administration of levodopa for relief of herpes zoster pain. J Am Med Assoc 1981; 246:132–4. 7017177

[21] Ertas M, Sagduyu A, Arac N, Uludag B, Ertekin C. Use of levodopa to relieve pain from painful symmetrical diabetic polyneuropathy. Pain 1998; 75:257–9. 9583761 10.1016/s0304-3959(98)00003-7

[22] Finnerup NB, Scholz J, Attal N, Baron R, Haanpää M, Hansson P, et al Neuropathic pain needs systematic classification. Eur J Pain. 2013 8; 17(7):953–6. doi: 10.1002/j.1532-2149.2012.00282.x 23339030 10.1002/j.1532-2149.2012.00282.x

[23] Finnerup NB, Otto M, McQuay HJ, Jensen TS, Sindrup SH. Algorithm for neuropathic pain treatment: an evidence based proposal. Pain. 2005 12 5; 118(3):289–305. doi: 10.1016/j.pain.2005.08.013 16213659 10.1016/j.pain.2005.08.013

[24] Attal N, Cruccu G, Haanpää M, et al EFNS guidelines on pharmacological treatment of neuropathic pain. Eur J Neurol. 2006 11; 13(11):1153–69. doi: 10.1111/j.1468-1331.2006.01511.x 17038030 10.1111/j.1468-1331.2006.01511.x

[25] Yin K, Zimmermann K, Vetter I, Lewis RJ. Therapeutic opportunities for targeting cold pain pathways. Biochem Pharmacol. 2015 1; 15;93(2):125–40. doi: 10.1016/j.bcp.2014.09.024 25316567 10.1016/j.bcp.2014.09.024

[26] Jørum E, Warncke T, Stubhaug A. Cold allodynia and hyperalgesia in neuropathic pain: the effect of N-methyl-D-aspartate (NMDA) receptor antagonist ketamine—a double-blind, cross-over comparison with alfentanil and placebo. Pain. 2003 2;101(3):229–35. 12583865 10.1016/S0304-3959(02)00122-7

[27] Treede RD, Jensen TS, Campbell JN, Cruccu G, Dostrovsky JO, Griffin JW, et al Neuropathic pain: redefinition and a grading system for clinical and research purposes. Neurology. 2008 4 29; 70(18):1630–5. doi: 10.1212/01.wnl.0000282763.29778.59 18003941 10.1212/01.wnl.0000282763.29778.59

[28] Ribarič S. The pharmacological properties and therapeutic use of apomorphine. Molecules 2012; 175: 5289–309. 10.3390/molecules17055289 PMC6268166 22565480

[29] Faul F1, Erdfelder E, Buchner A, Lang AG. Statistical power analyses using G*Power 3.1: tests for correlation and regression analyses. Behav Res Methods. 2009 11;41(4):1149–60. doi: 10.3758/BRM.41.4.1149 19897823 10.3758/BRM.41.4.1149

[30] Treister R, Eisenberg E, Demeter N, Pud D. Alterations in pain response are partially reversed by methylphenidate (ritalin) in adults with attention deficit hyperactivity disorder (ADHD). Pain Pract. 2015 1; 15(1):4–11. doi: 10.1111/papr.12129 24134430 10.1111/papr.12129

[31] Tuttle AH1, Tohyama S, Ramsay T, Kimmelman J, Schweinhardt P, Bennett GJ, Mogil JS. Increasing placebo responses over time in U.S. clinical trials of neuropathic pain. Pain. 2015 12;156(12):2616–26 doi: 10.1097/j.pain.0000000000000333 26307858 10.1097/j.pain.0000000000000333

[32] Charbit AR, Akerman S, Goadsby PJ. Comparison of the effects of central and peripheral dopamine receptor activation on evoked firing in the trigeminocervical complex. J Pharmacol Exp Ther 2009; 331(2):752–63. doi: 10.1124/jpet.109.151951 19657051 10.1124/jpet.109.151951

[33] Main DC, Waterman AE, Kilpatrick IC, Jones A. An assessment of the peripheral antinociceptive potential of remoxipride, clonidine and fentanyl in sheep using the forelimb tourniquet. J Vet Pharmacol Ther 1997; 20(3):220–8. 9185089 10.1111/j.1365-2885.1997.tb00099.x

[34] Reinersmann A, Maier C, Schwenkreis P, Lenz M. Complex regional pain syndrome: more than a peripheral disease. Pain Manag. 2013 11; 3(6):495–502. doi: 10.2217/pmt.13.53 24654904 10.2217/pmt.13.53

[35] Bromberg-Martin ES, Matsumoto M, Hikosaka O. Dopamine in motivational control: rewarding, aversive, and alerting. Neuron 2010; 68(5):815–34. doi: 10.1016/j.neuron.2010.11.022 21144997 10.1016/j.neuron.2010.11.022 PMC3032992

[36] Cools R. Role of dopamine in the motivational and cognitive control of behavior. Neuroscientist 2008; 14(4):381–95. doi: 10.1177/1073858408317009 18660464 10.1177/1073858408317009

[37] Salamone JD. The behavioral neurochemistry of motivation: methodological and conceptual issues in studies of the dynamic activity of nucleus accumbens dopamine. J Neurosci Methods 1996; 64(2):137–49. 8699874 10.1016/0165-0270(95)00125-5

[38] Salamone JD, Correa M, Farrar A, Mingote SM. Effort-related functions of nucleus accumbens dopamine and associated forebrain circuits. Psychopharmacology (Berl) 2007; 191(3):461–82. 17225164 10.1007/s00213-006-0668-9

[39] de la Fuente-Fernandez R, Schulzer M, Stoessl AJ. The placebo effect in neurological disorders. Lancet Neurol 2002; 1:85–91. 12849512 10.1016/s1474-4422(02)00038-8

[40] de la Fuente-Fernández R, Phillips A.G, Zamburlini M, Sossi V, Calne D.B, Ruth T.J and Stoessl A.J. Dopamine release in human ventral striatum and expectation of reward. Behavioural Brain Research 2002; 136: 359–363. 12429397 10.1016/s0166-4328(02)00130-4

[41] Benedetti F. How the doctor’s words affect the patient’s brain. Evaluation & the Health Professions 2002; 25: 369–386. 12449081 10.1177/0163278702238051

[42] de la Fuente-Fernández R. The placebo-reward hypothesis: dopamine and the placebo effect. Parkinsonism Relat Disord. 2009 12; 15 Suppl 3:S72–4. 20083013 10.1016/S1353-8020(09)70785-0

[43] Petrovic P, Kalso E, Petersson KM, Ingvar M. Placebo and opioid analgesia–imaging a shared neuronal network. Science 2002; 295:1737–40. doi: 10.1126/science.1067176 11834781 10.1126/science.1067176

[44] Scott DJ, Stohler CS, Egnatuk CM, Wang H, Koeppe RA, Zubieta JK. Individual differences in reward responding explain placebo-induced expectations and effects. Neuron 2007; 55:325–36. doi: 10.1016/j.neuron.2007.06.028 17640532 10.1016/j.neuron.2007.06.028

[45] dela Peña 1, Gevorkiana R, Shi WX. Psychostimulants affect dopamine transmission through both dopamine transporter-dependent and independent mechanisms. Eur J Pharmacol. 2015 10 5;764:562–70. doi: 10.1016/j.ejphar.2015.07.044 26209364 10.1016/j.ejphar.2015.07.044 PMC4600454

[46] Aarsland D, Pahlhagen S, Ballard CG, Ehrt U, Svenningsson P. Depression in Parkinson disease–epidemiology, mechanisms and management. Nat Rev Neurol. 2011; 8(1):35–47. doi: 10.1038/nrneurol.2011.189 22198405 10.1038/nrneurol.2011.189

[47] Bair MJ, Robinson RL, Katon W, Kroenke K. Depression and pain comorbidity: a literature review. Arch Intern Med. 2003 11 10;163(20):2433–45. doi: 10.1001/archinte.163.20.2433 14609780 10.1001/archinte.163.20.2433

[48] Floresco SB, West AR, Ash B, Moore H, Grace AA. Afferent modulation of dopamine neuron firing differentially regulates tonic and phasic dopamine transmission. Nat Neurosci 2003; 6(9):968–73. doi: 10.1038/nn1103 12897785 10.1038/nn1103

[49] Grace AA. Phasic versus tonic dopamine release and the modulation of dopamine system responsivity: a hypothesis for the etiology of schizophrenia. Neuroscience 1991; 41(1):1–24. 1676137 10.1016/0306-4522(91)90196-u

[50] Leknes S, Tracey I. A common neurobiology for pain and pleasure. Nat Rev Neurosci 2008; 9(4):314–20. doi: 10.1038/nrn2333 18354400 10.1038/nrn2333

[51] de La Fuente-Ferna´ndez R, Lim AS, Sossi V, Holden JE, Calne DB, et al Apomorphine-induced changes in synaptic dopamine levels: positron emission tomography evidence for presynaptic inhibition. J Cereb Blood Flow Metab 2001; 21(10):1151–9. doi: 10.1097/00004647-200110000-00003 11598492 10.1097/00004647-200110000-00003

[52] Przedborski S, Levivier M, Raftopoulos C, Naini AB, Hildebrand J Peripheral and central pharmacokinetics of apomorphine and its effect on dopamine metabolism in humans. Mov Disord 1995; 10(1):28–36. doi: 10.1002/mds.870100107 7885353 10.1002/mds.870100107

[53] Gilron I, Jensen TS, Dickenson AH. Combination pharmacotherapy for management of chronic pain: from bench to bedside. Lancet Neurol 2013; 12,1084–95. doi: 10.1016/S1474-4422(13)70193-5 24074723 10.1016/S1474-4422(13)70193-5

