# Supplementary material for: The effects of a dopamine agonist (apomorphine) on experimental and spontaneous pain in patients with chronic radicular pain: A randomized, double-blind, placebo-controlled, cross-over study
Source: PLoS One. 2018 Apr 5;13(4):e0195287. doi: 10.1371/journal.pone.0195287 (PMC5886417; doi:10.1371/journal.pone.0195287)
Supplement: S1 Text — (PDF) [file pone.0195287.s002.pdf]

Analgesic response to dopamine agonists in patients with neuropathic pain

1. *Introduction*

1.1. Pain

One of the vital functions of the nervous system is to provide information about the occurrence of threat or injury. The sensation of pain by being an alarming mechanism contributes to this function [1].

The International Association for the study of Pain (IASP) defines pain as "An unpleasant sensory and emotional experience associated with actual or potential tissue damage, or described in terms of such damage"[2]. What makes the experience of pain special compared to senses is the profound emotional quality of the pain experience in addition to its physiological and pathophysiological elements. Furthermore, according to this definition, pain is a subjective experience, which is associated with either actual or potential harm.

1.2. Neuropathic pain

Neuropathic pain (NP) is defined as "pain arising as a direct consequence of a lesion or disease affecting the somatosensory nervous system"[3]. Hence, NP may result from disease or lesion to either the central or the peripheral nervous systems. Common examples of peripheral NP include lumbar or cervical radiculopathy, painful diabetic neuropathy (PDN) and post-herpetic neuralgia (PHN). NP of central origin includes central post-stroke pain, pain due to multiple sclerosis, and post-spinal cord injury pain. From the etiological standpoint, NP can result from trauma, inflammation, infection, toxicity, metabolic disorders etc.

NP may be continuous or intermittent, and is typically described by patients as burning, aching, or shooting. Allodynia (pain due to a stimulus that does not normally provoke pain), hyperalgesia (an increased response to a stimulus that is normally painful) and paresthesia (an abnormal sensation whether spontaneous or evoked) are commonly associated with NP [4, 5].

An estimated 1.5%-8% of the general populations suffer from NP [6]. It is estimated to affect millions of people worldwide. The prevalence of different types of NP varies widely [7]. NP is associated with high degree of suffering and can be severe and disabling, with significant functional, psychological, and social consequences. It is often undiagnosed and undertreated [8] and therefore it is considered to be one of the greatest challenges among pain relief health professionals.

1.3. Neuropathic pain treatments

Appropriate diagnosis and assessment are critical to the successful treatment of NP. The diagnosis of NP can often be challenging, partially because it commonly coexists with other types of pain, as well as with other symptoms like depression, anxiety, sleep disturbances, and other adverse impacts on health-related quality of life [9, 10].

The management of patients with chronic NP is complex and the response to commonly existing treatments is often inadequate: effective pain relief occurs in less than half of NP patients [11, 12]. Although many different drug groups are used to manage NP, choosing the "right therapy" is often challenging because of the unpredictable effectiveness, complicated dosing regimens, delayed analgesic onset and common side effects.

Pharmacotherapy for NP is not necessarily based on common analgesic medication (e.g., opioids), and includes antidepressants, anticonvulsants, and topical agents [13, 14]. Additional approaches for the management of intractable NP include implantation of neuromodulation devices such as spinal cord stimulators or intrathecal drug delivery pumps. Anecdotal reports also suggest efficacy of additional therapies such as NMDA receptor antagonists and dopamine agonists.

1.4. Dopamine

Dopamine is a monoamine neurotransmitter best known for its role in movement and cognition. The role of dopamine in human pathology is evident in disorders such as Parkinson's disease (PD), schizophrenia and addiction.

There are five subtypes of dopamine receptors, designated D1 through D5 [15]. The D1 and D5 receptors are members of the D1 subfamily of dopamine receptors, whereas the D2, D3 and D4 receptors comprise the D2 subfamily. D1 receptors are expressed throughout the brain and spinal cord [16]. The highest densities occur in the basal ganglia and substantia nigra (SN). Dopamine D2 receptors are also expressed throughout the brain with highest concentration in the basal ganglia, globus pallidus, SN and ventral tegmental area (VTA) [17]. D3 are expressed in the basal ganglia, SN, globus pallidus and in anterior and medial thalamic nuclei. They are also present in the limbic cortex, locus coeruleus and raphe nuclei [18]. Dopamine D5 receptors are localized in the SN, cerebral cortex and basal ganglia [19].

Dopaminergic neurotransmition demonstrated a central role in modulating pain perception and natural analgesia within supraspinal regions including the basal ganglia [20], insula [21], anterior cingulated cortex [22], and periaquaductal gray [23] as well as within the spinal cord

[24]. Furthermore, a critical role of dopamine in descending inhibition has also been demonstrated [24, 25, 26].

1.5. Chronic pain in conditions with dopamine deficiency

Parkinson's disease results from degeneration of dopamine neurons. It is a movement disorder but patients with Parkinson’s disease are known to be more sensitive to pain than healthy subjects [27]. Decreased levels of dopamine likely contribute to the painful symptoms that frequently occur in Parkinson's disease [28, 29].

Burning mouth syndrome (BMS) is a chronic orofacial pain disorder characterized by burning sensations in the tongue or other oral sites that are frequently accompanied by dryness and taste alterations. Chronic orofacial pain conditions are associated with alterations in the striatal dopaminergic system characterized by reduced presynaptic activity of striatal dopamine neurons and changes in dopamine D2 receptor availability [29, 30]. Abnormalities in dopaminergic neurotransmission have been demonstrated in additional painful clinical conditions, including Fibromyalgia syndrome (FMS) which is a complex disorder of chronic widespread pain, fatigue, and sleep disturbances [31] as well as restless legs syndrome (RLS), a disorder characterized by unpleasant leg sensations together with an irresistible inner urge to move. Symptoms of RLS are worsened at rest and in the evening, leading to insomnia and daytime sleepiness [32].

The relationship between dopamine and pain is further strengthened by the fact that several medications with dopaminergic properties have also been found to display analgesic activity. For example, amphetamine is an indirect dopamine agonist that acts to increase the release of dopamine from neuron terminals while simultaneously inhibiting its reuptake by the dopamine transporter. Studies have demonstrated that the analgesic capacity of amphetamine is predicated on its impact on supraspinal dopamine D2 receptor binding [20]. Other examples include cocaine [33, 34] and cannabinoids [35, 36].

The administration of L-DOPA to patients with Parkinson’s disease has been shown to result in an increase in pain thresholds [37, 38, 39]. A controlled study demonstrated the efficacy of Pramipexole (a dopamine agonist) in the treatment of Fibromyalgia syndrome [40]. In this 14-week, double blind, placebo-controlled, parallel-group, escalating dose trial, 60 patients with fibromyalgia were randomized to receive 4.5 mg of pramipexole or placebo orally every evening. Compared with the placebo group, patients receiving pramipexole experienced gradual and more significant improvement in pain, fatigue and function. In addition, a few

controlled trails demonstrated short-term efficacy and safety of L-DOPA for restless legs syndrome [41].

1.6. Efficacy of dopaminergic medications for neuropathic pain

A small number of clinical trials suggest that medications with dopaminergic activity can possibly reduce pain in patients with various forms of NP such as bone metastases [42]. In one study, 47 patients with herpes zoster received oral levodopa and benserazide or placebo in a double-blind controlled study for ten days. A significant decrease in pain intensity was seen in the group receiving levodopa from the third day, as well as a complete cessation of both pain and sleep disturbances was more frequent in these patients [43]. In addition, a double-blind placebo controlled study was conducted and included patients with painful diabetic neuropathies who suffer from distal burning pains in the extremities [44]. All patients were not taking any medication for pain. Fourteen patients were given 100 mg levodopa plus 25 mg benserazide capsules three times per day for 28 days. Eleven patients were given identical placebo capsules. A blinded neurologist evaluated the patients clinically and performed Visual Analogue Scale (VAS, 0-10) measurement every week from day 0 to day 28. From week 2 to week 4, the VAS scores of the active drug group decreased significantly compared to the placebo group. According to these results it was suggested to use levodopa for pain control in neuropathy. Yet, no firm conclusions regarding the efficacy of dopaminergic agents for the treatment of NP can be drawn from these limited small samples sized studies. There is a need for further controlled trails to study the efficacy of medications with dopaminergic properties when treating NP.

1.10. Preliminary results

In an attempt to bridge the gap between animal studies to clinical trials, we have explored in our laboratory the effect of apomorphine injection on experimental pain in a sample of healthy volunteers. So far, data were collected from 61 subjects. Each subject participated in two sessions and received in a randomized double blind manner: apomorphine injection in one session and placebo in the other. The subjects were exposed to cold painful stimuli before and after drug administration. Apomorphine, but not placebo, significantly increased the time to hand withdrawal from ice water one hour following drug administration ($p = 0.033$). This finding indicates that doapminergic intervention prolongs tolerance to cold pain.

1.11. Aims of the Study

1. To determine the effect of dopaminergic intervention on experiemantal pain in patients with neuropathic pain.
2. To determine the effect of dopaminergic intervention on spontaneous and evoked neuropathic pain in patients with this type of pain.

1.12. Significance of the study

The results of the proposed study can shed more light on the role of dopamine in pain processing and potentially contribute to the development of new treatments for neropathic pain.

2. Methods

2.1. Participants

A sample of 100 men and women with chronic neuropathic pain, aged 18-75, will be enrolled in this study after meeting the following inclusion criteria:

1) Adults who are capable of understanding the purpose and instructions of the study and signing an informed consent.

2) Presence of neuropathic pain for at least three months.

3) Average pain level during the last week prior to enrollment $\geq$ 4 (0-10).

4) No use of a new analgesic drug within 30 days prior to entry to the study (patients will continue using their regular analgesic medications).

Patients will be excluded from the study if they will meet one of the following exclusion criteria:

1) Pregnant or breastfeeding women.

2) Presence of Parkinson's disease or any other extra-pyramidal diseases.

3) History of allergy to the investigational drugs: Apomorphine or Motilium.

4) History of polyneuropathy.

5) Patients with respiratory depression, dementia, psychotic diseases or hepatic insufficiency.

2.2. Instruments

2.2.1. **Cold pressor test apparatus (CPT)**

The CPT apparatus (Heto CBN 8-30 Lab equipment, Allerod, Denmark) is a temperature-controlled water bath with a maximum temperature variance of ± 0.5°C, which is continuously stirred by a pump.

2.2.2. A numerical pain scale (NPS)

The NPS is a scale ranging from 0 = ‘‘no pain” to 100 = ‘‘the worst pain one can imagine”, will be verbally used by patients to grade the magnitude of their current spontaneous neuropathic pain during each session.

2.2.3. Questionnaires

LANSS pain scale: the Leeds Assessment of Neuropathic Symptoms and Signs

The LANSS contains 5 symptom items and 2 clinical examination items, and is easy to score within clinical settings [45]. The LANSS has been tested and validated in several settings with high sensitivity and specificity compared to clinical diagnosis [46, 47]. Positive scores on the LANSS identify patients with pain of predominantly neuropathic origin. This pain scale provides immediate information in a clinical setting.

Douleur Neuropathique en 4 questions (DN4)

DN4 consists of four questions with seven items related to symptoms and 3 related to clinical examination [48]. The DN4 is easy to score and the maximal score is 10. A total score of 4 out of 10 or more suggests neuropathic pain. The DN4 showed high sensitivity and specificity when compared to clinical diagnosis [48]. The questionnaire was translated and validated into different languages, including Hebrew.

2.3. Pain tests

2.3.1 Assessment of cold pain perception in the hand

Patients will be asked to place their dominant hand in the CPT apparatus (12°C) in a still position with their fingers spread wide apart. A stopwatch will be simultaneously activated, and patients will be requested to maintain their hand in the cold water for as long as they can. They will be instructed to indicate the exact point in time when the cold sensation begins to elicit pain. This time until the pain is first perceived will be defined as the threshold of cold

pain (seconds). A cut-off time of 180 seconds will be set for safety reasons. Time until spontaneous hand withdrawal will be also recorded and defined as cold pain tolerance (seconds). Immediately after hand withdrawal, subjects will be asked to rate their maximal pain intensity using the NPS scale (0-100).

2.3.2. Assessment of cold pain in the most painful site using ice

A flexile ice pack (25 cm X 17 cm) will be placed on the area with maximal pain in the affected leg. A stopwatch will be simultaneously activated, and patients will be requested to keep the ice pack on the leg as long as they can. The patients will be instructed to indicate the exact time point when the cold sensation begins to elicit pain. This time will be defined as the of cold pain threshold (seconds). Time until the ice pack is not tolerated anymore and removed will be also recorded and defined as pain tolerance (seconds). A cut-off time of 180 seconds is set for safety reasons. Immediately after the ice pack removal, the subjects will be asked to rate their maximal pain intensity using the NPS scale (0-100).

2.4. Study medications

The study medications are:

- A single dose of apomorphine, an injectable, potent, short-acting dopamine agonist. Based on a previous study, conducted in our laboratory, 1.5 mg apomorphine was determined to be the appropriate dose for this study.
- An identical looking placebo (saline).
- Domperidone: most of apomorphine side effects can be sufficiently reduced with a preparation of domperidone, a peripheral dopamine antagonist. The subjects will be instructed to take domperidone (10 mg, oral) three times a day for three days preceding both study medications.

Apomorphine and the identical looking placebo (saline) syringes will be prepared and injected by a nurse according to a pre-determined randomization.

2.5. Study Design

Patients with NP will be enrolled in this study if they meet the inclusion/ exclusion criteria. Each patient will receive detailed information about the study and the study procedures including the fact that at each session one of two possible study medications (apomorphine /

placebo) will be administered in a blinded fashion. If the patient decides to participate, he or she will be asked to sign an informed consent form. Each patient will participate in two sessions, each will last approximately 3 hours, one week apart. Participants will be asked to take a total of 10 x 1mg Mutilium tablets, 3 times a day, for 3 days before each session.

During the first session patients will be asked to fill the LANSS pain scale (partially by the patient and partially by the physician) and the questionnaire for quick diagnosis of neuropathic pain (DN4). Patients will then be exposed to the following tests: cold pain threshold and tolerance in the hand; cold pain threshold and tolerance in the most painful site in the affected leg. They will be also asked to report their current spontaneous neuropathic pain (NPS 0-100). The first round of pain tests will be considered as training and its results will not be used in the statistical analyses. Fifteen minutes later a second round of tests will be conducted and the results will be recorded and regarded as baseline measurements. Each patient will then receive a S.C. injection of either 1.5 mg apomorphine or a placebo in a double blind fashion. Three additional pain tests rounds will be conducted 30, 75 and 120 minutes after drug administration. During each session, patients will be requested to record any adverse effect and to grade them on a 0–3 scale, where 0 = none, 1 = mild, 2 = moderate, and 3 = severe. One week later the patient will be asked to arrive for a second session that will be performed in the same manner but without the saliva sample and with the other drug (apomorphine or placebo).

2.6. Statistical analysis

Analyses will be conducted using the SPSS for Windows Version 17 statistical package (SPSS, Inc., Chicago, IL). The differences between the analgesic effects of the study medication, compared to placebo, on experimental pain and neuropathic pain will be analyzed by a repeated measure ANOVA for each pain measure separately. The relations between the analgesic effect of the study medication on experimental and neuropathic pain will be examined using Person's correlation tests.

References

1. Wall, P.D. and Melzack, R. Text book of pain. 5th ed. (2006).
2. Merskey H, Bogduk N, eds. 1994. Classification of Chronic Pain, 2nd ed. Seattle, WA: IASP Press. 209-214.
3. Treede RD, Jensen TS, Campbell JN, Cruccu G, Dostrovsky JO, Griffin JW, Hansson P, Hughes R, Nurmikko T, Serra J. Neuropathic pain: redefinition and a grading system for clinical and research purposes. Neurology. 2008 Apr 29;70(18):1630-5.
4. Turk D.C, Okifuji A. 2010. Pain Terms and Taxonomies. in Fishman, S.M..; Ballantyne, J.C.; Rathmell, J.P. et al. Bonica's management of pain (4 edn). pp. 13–23.
5. Hansson P, Laceremza M, Marchettini P. Aspects of clinical and experimental neuropathic pain: The clinical perspective. In: Hansson P, Fields HL, Hill RG, Marchettini P, eds. Neuropathic Pain: Pathophysiology and Treatment. Vol 21. Seattle: IASP Press; 2001:1-18.
6. Hall GC, Carroll D, Parry D, McQuay HJ. Epidemiology and treatment of neuropathic pain: the UK primary care perspective. Pain. 2006 May;122(1-2):156-62.
7. Bennett GJ. Neuropathic pain: new insights, new interventions. Hosp Pract (Minneap). 107-10 passim, 1998 Oct 15 1998;33(10):95-98.
8. Taylor RS. Epidemiology of refractory neuropathic pain. Pain Pract. 2006 Mar; 6(1):22-6.
9. Jensen MP, Chodroff MJ, Dworkin RH. The impact of neuropathic pain on health-related quality of life: review and implications. Neurology 2007;68:1178–82.
10. Meyer-Rosberg K, Kvarnstro¨m A, Kinnman E, Gordh T, Nordfors LO, Kristofferson A. Peripheral neuropathic pain: a multidimensional burden for patients. Eur J Pain 2001;5:379–89
11. Finnerup NB, Otto M, McQuay HJ, Jensen TS, Sindrup SH. Algorithm for neuropathic pain treatment: an evidence based proposal. Pain. 2005 Dec 5;118(3):289-305.
12. Attal N, Cruccu G, Haanpää M, et al. EFNS guidelines on pharmacological treatment of neuropathic pain. Eur J Neurol. 2006 Nov;13(11):1153-69.
13. Dworkin RH, O'Connor AB, Backonja M, Farrar JT, Finnerup NB, Jensen TS, Kalso EA, Loeser JD, Miaskowski C, Nurmikko TJ, Portenoy RK, Rice AS, Stacey BR, Treede RD, Turk DC, Wallace MS. Pharmacologic management of neuropathic pain: evidence-based recommendations. Pain. 2007;132(3):237-51**.**
14. Eisenberg E, Peterson D. Neuropathic pain pharmacotherapy. in Fishman, S.M..; Ballantyne, J.C.; Rathmell, J.P. et al. Bonica's management of pain (4 edn). 2010. 1194–1204.
15. Sibley DR, Monsma FJ Jr, Shen Y. Molecular neurobiology of dopaminergic receptors. Int. Rev. Neurobiol. 1993; 35: 391-415.
16. Wamsley JK, Alburges ME, McQuade RD, Hunt M. CNS distribution of D1 receptors: use of a new specific D1 receptor antagonist, [3H]SCH39166. Neurochem Int. 1992; 20(Suppl.): S123-S128.
17. Gurevich EV, Joyce JN. Distribution of dopamine D3 receptor expressing neurons in the human forebrain: comparison with D2 receptor expressing neurons. Neuropsychopharmacology. 1999; 20(1): 60-80.

18. Suzuki M, Hurd YL, Sokoloff P, Schwartz JC, Sedvall G. D3 dopamine receptor mRNA is widely expressed in the human brain. Brain Res. 1998; 779(1-2): 58-74.

19. Khan ZU, Gutiérrez A, Martín R, Peñafiel A, Rivera A, de la Calle A. Dopamine D5 receptors of rat and human brain. Neuroscience 2000; 100(4): 689-699.

20. Alteir N, Stewart J. The role of dopamine in the nucleus accumbens in analgesia. Life Sci. 1999;65(22):2269-87.

21. Burkey AR, Carstens E, Jasmin L. Dopamine reuptake inhibition in the rostral agranular insular cortex produces antinociception.J Neurosci. 1999; 19(10):4169-79.

22. Lopez-Avila A, Coffeen U, Ortega-Legaspi JM, del Angel R, Pellicer F. Dopamine and NMDA systems modulate long-term nociception in the rat anterior cingulated cortex. Pain. 2004;111 (1-2):136-43.

23. Flores JA, El Banoua F, Galan-Rodriguez B, Fernandez-Espejo E. Opiate antinociception is attenuated following lesion of large dopamine neurons of the periaqueductal grey: critical role for D1 (not D2) dopamine receptors. Pain. 2004;110(1-2):205-14.

24. Lindvall O, Bjorklund A, Skagerberg G. Dopamine-containing neurons in the spinal cord: anatomy and some functional aspects. Ann Neurol. 14(3):255-60, 1983.

25. Wood PB. Role of central dopamine in pain and analgesia. Expert Rev Neurother 2008;8:781–97.

26. Wei H, Viisanen H, Pertovaara A. Descending modulation of neuropathic hypersensitivity by dopamine D2 receptors in or adjacent to the hypothalamic A11 cell group. Pharmacol Res. 2009;59(5):355-63.

27. Tinazzi M, Del Vesco C, Fincati E, Ottaviani S, Smania N, Moretto G, Fiaschi A, Martino D, Defazio G. Pain and motor complications in Parkinson’s disease. J Neurol Neurosurg Psychiatry 2006;77:822–5.

28. Defazio G, Berardelli A, Fabbrini G, Martino D, Fincati E, Fiaschi A, Moretto G, Abbruzzese G, Marchese R, Bonuccelli U, Del Dotto P, Barone P, De Vivo E, Albanese A, Antonini A, Canesi M, Lopiano L, Zibetti M, Nappi G, Martignoni E, Lamberti P, Tinazzi M. Pain as a nonmotor symptom of Parkinson disease: evidence from a case-control study. Arch Neurol. 2008 Sep;65(9):1191-4.

29. Jaaskelainen SK, Rinne JO, Forssell H, Tenovuo O, Kaasinen V, Sonninen P, Bergman J. Role of the dopaminergic system in chronic pain - a fluorodopa-PET study. Pain. 2001; 90(3):257-60.

30. Hagelberg N, Forssell H, Rinne JO et al. Striatal dopamine D1 and D2 receptors in burning mouth syndrome. Pain. 2003; 101(1-2): 149-154.

31. Wood PB, Patterson JC 2nd, Sunderland JJ, Tainter KH, Glabus MF, Lilien DL. Reduced presynaptic dopamine activity in fibromyalgia syndrome demonstrated with positron emission tomography: a pilot study. J Pain. 2007; 8(1):51-8.

32. Cervenka S, Pålhagen SE, Comley RA, Panagiotidis G, Cselényi Z, Matthews JC, Lai RY, Halldin C, Farde L.Support for dopaminergic hypoactivity in restless legs syndrome: a PET study on D2-receptor binding. Brain. 2006 Aug;129 (Pt 8):2017-28.

33. Shyu BC, Kiritsy-Roy JA, Morrow TJ, Casey KL. Neurophysiological, pharmacological and behavioral evidence for medial thalamic mediation of cocaine-induced dopaminergic analgesia. Brain Res 1992;572:216– 223.

34. Lin Y, Morrow TJ, Kiritsy-Roy JA, Terry LC, Casey KL. Cocaine: evidence for supraspinal, dopamine-mediated, non-opiate analgesia. Brain Res. 1989 Feb 13;479(2):306-12.

35. Fadda P, Scherma M, Spano MS, Salis P, Melis V, Fattore L, Fratta W Neuroreport. Cannabinoid self-administration increases dopamine release in the nucleus accumbens. 2006 Oct 23;17(15):1629-32.

36. Carta G, Gessa GL, Nava F. Eur J Pharmacol. Dopamine D(2) receptor antagonists prevent delta(9)-tetrahydrocannabinol-induced antinociception in rats. 1999 Nov 19;384(2-3):153-6.

37. JL, Rascol O. Effect of levodopa on pain threshold in Parkinson’s disease: a clinical and positron emission tomography study. Mov Disord 2005;20:1557–63.

38. Gerdelat-Mas A, Simonetta-Moreau M, Thalamas C, Ory-Magne F, Slaoui T, Rascol O, Brefel-Courbon C. Levodopa raises objective pain threshold in Parkinson’s disease: a RIII reflex study. J Neurol Neurosurg Psychiatry 2007;78(10):1140–2.

39. Slaoui T, Mas-Gerdelat A, Ory-Magne F, Rascol O, Brefel-Courbon C. Levodopa modifies pain thresholds in Parkinson’s disease patients. Rev Neurol (Paris) 2007;163:66–71.

40. Holman AJ, Myers RR. A randomized, double-blind, placebo-controlled trial of pramipexole, a dopamine agonist, in patients with fibromyalgia receiving concomitant medications. Arthritis Rheum 2005;52:2495–505.

41. Conti CF, de Oliveira MM, Andriolo RB, Saconato H, Atallah AN, Valbuza JS, Coin de Carvalho LB, do Prado GF. Levodopa for idiopathic restless legs syndrome: evidence-based review. Mov Disord. 2007 Oct 15;22(13):1943-51. Review.

42. Dickey RP, Minton JP. Levodopa relief of bone pain from breast cancer. N Engl J Med 1972;286:843.

43. Kernbaum S, Hauchecorne J. Administration of levodopa for relief of herpes zoster pain. J Am Med Assoc 1981;246:132–4.

44. Ertas M, Sagduyu A, Arac N, Uludag B, Ertekin C. Use of levodopa to relieve pain from painful symmetrical diabetic polyneuropathy. Pain 1998;75:257–9.

45. Bennett MI. The LANSS Pain Scale: the Leeds assessment of neuropathic symptoms and signs. Pain 2001;92:147–57.

46. Potter J, Higginson IJ, Scadding JW, Quigley CW. Identifying neuropathic pain in patients with head and neck cancer: use of the Leeds Assessment of Neuropathic Symptoms and Signs Scale. J R Soc Med. 2003;96:379–83.

47. Yucel A, Senocak M, Kocasoy Orhan E, Cimen A, Ertas M. Results of the Leeds assessment of neuropathic symptoms and signs pain scale in Turkey: a validation study. J Pain 2004;5:427–32.

48. Bouhassira D, Attal N, Alchaar H, Boureau F, Bruxelle J, Cunin G, et al. Comparison of pain syndromes associated with nervous or somatic lesions and development of a new neuropathic pain diagnostic questionnaire (DN4). Pain 2005;114:29–36.
